# A Cu-bis(imidazole) Substrate Intermediate Is the Catalytically Competent Center for Catechol Oxidase Activity of Copper Amyloid-β

**DOI:** 10.1021/acs.inorgchem.0c02243

**Published:** 2021-01-06

**Authors:** Chiara Bacchella, Simone Dell’Acqua, Stefania Nicolis, Enrico Monzani, Luigi Casella

**Affiliations:** Dipartimento di Chimica, Università di Pavia, Via Taramelli 12, Pavia 27100, Italy

## Abstract

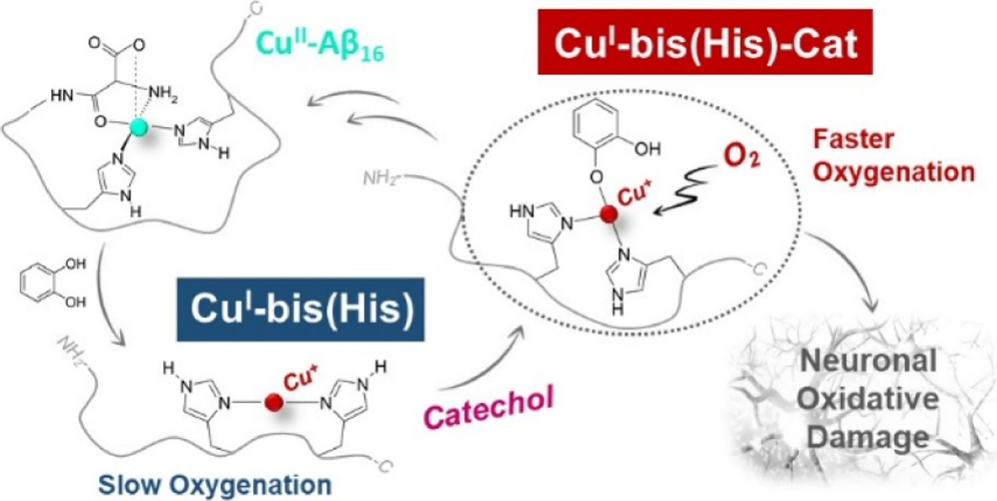

Interaction of copper ions with Aβ peptides alters the redox activity of the metal ion and can be associated with neurodegeneration. Many studies deal with the characterization of the copper binding mode responsible for the reactivity. Oxidation experiments of dopamine and related catechols by copper(II) complexes with the N-terminal amyloid-β peptides Aβ_16_ and Aβ_9_, and the Aβ_16_[H6A] and Aβ_16_[H13A] mutant forms, both in their free amine and N-acetylated forms show that efficient reactivity requires the oxygenation of a Cu^I^-bis(imidazole) complex with a bound substrate. Therefore, the active intermediate for catechol oxidation differs from the proposed “in-between state” for the catalytic oxidation of ascorbate. During the catechol oxidation process, hydrogen peroxide and superoxide anion are formed but give only a minor contribution to the reaction.

## Abstract 2

The redox cycling of copper bound to amyloid-β peptide requires the generation of a Cu(I)-Aβ-catecholate complex. When copper(II) is confined in the N-terminal portion, its reduction is slow and causes a shift toward a bis-His coordination environment. The addition of catechol to the Cu(I)-bis(imidazole) complex results in a faster reaction with dioxygen. The reactive species for catechol oxidation does not correspond to the proposed “in-between state” for ascorbate oxidation.

## Introduction

Alzheimer’s disease (AD) is a neurodegenerative disorder characterized by the deposition of amyloid-β (Aβ) into the extracellular “senile plaques”,^1^ and by the presence of intracellular neurofibrillary tangles of β-folded tau.^2,3^ In addition to amyloidosis, the dyshomeostasis of redox-active metals promotes the fast disease progression; in particular, higher levels of extracellular labile copper compared to a normal brain have been observed in AD brains.^4,5^ A strong correlation between the binding of metal ions with Aβ and the cascade of events resulting in neuronal damage was suggested.^6,7^ In particular, copper redox activity seems to play an important role in this process.^8−10^

Aβ peptides are generated by proteolytic cleavage of the amyloid precursor protein, the major species being those containing 40 and 42 residues.^11^ All isoforms exhibit an unstructured N-terminal region, able to bind Cu^II^/Cu^I^ ions at physiological pH through a dynamic equilibrium between several coexisting binding modes. The main Cu^II^ species at neutral pH, called “component I”, contains the NH_2_ and C=O groups of Asp1, and the imidazole groups of His6, and either His13 or His14 as ligands.^12^ For the minor “component II”, the ligand set comprises the NH_2_ of Asp1, the deprotonated amide group between Asp1 and Ala2, the C=O group of Ala2, and the imidazole group of one of the histidines.^13^ A linear coordination mode with two imidazole groups from His6, His13, and His14 has been identified as the major form for the Cu^I^-Aβ complex.^14^

On the other hand, the redox chemistry of these complexes does not seem compatible with the large structural rearrangement, and the energetic cost required for coupling the preferred Cu^II^ and Cu^I^ equilibrium forms. A highly reactive intermediate, called “catalytic in-between state”, has been proposed as a transition model able to minimize the energetic request in the ascorbate catalytic oxidation and involves the N-terminal amine, the side chain of Asp1, and the imidazole group of one histidine^8,15,16^ (probably H6).^17^ It would be interesting to know if this species can be competent to promote oxidation of other substrates, proteins, or lipids typically associated with AD.^18,19^

Among the various factors contributing to neurodegeneration, the trafficking and biochemical pathways of catecholamine neurotransmitters should be considered. Indeed, the reactivity of catecholamines toward transition metals promotes the generation of reactive species capable of modifying biomolecules, exacerbating neuronal tissue damage.^20^ This problem can be particularly relevant in the locus coeruleus, which is relatively rich in copper,^21,22^ and where Cu dyshomeostasis can interfere with the catecholamine metabolism and impair the functioning of noradrenergic neurons.^23^ In this paper, we aim at testing whether the “in-between” model proposed for the oxidation of ascorbate can be assumed as reliable catalytic species in the oxidation of catechols such as dopamine (DA). To this end, we investigated the reactivity of Cu^II^ complexes with Aβ_16_,^12,13^ the Aβ_16_[H6A] and Aβ_16_[H13A] mutants, and Aβ_9_ peptides^24^ in both unprotected (NH_2_-Aβ) and N-acetylated (Ac-Aβ) forms.

## Results and Discussion

**Figure 1:**
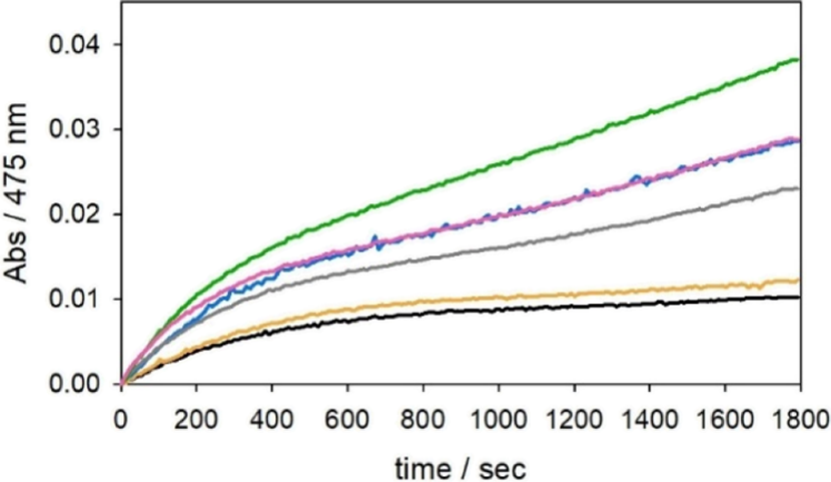
Kinetic profiles of DA (3 mM) oxidation with time in 50 mM HEPES buffer at pH 7.4 and 20 °C (autoxidation, black trace) in the presence of Cu^II^ (25 μM) (orange) and with 1 equiv NH_2_-Aβ_16_ (blue), 2 equiv NH_2_-Aβ_16_ (green), 1 equiv Ac-Aβ_16_ (grey), and 2 equiv Ac-Aβ_16_ (pink).

An initial experiment carried out to compare the oxidation of DA and 4-methylcatechol (MC) promoted by Cu^II^-NH_2_-Aβ_16_ and Cu^II^-Ac-Aβ_16_ (25–50 μM) at the saturating substrate concentration (3 mM) gave contradictory results (Figures 1 and S1). As in our previous papers,^20,25,26^ here, we follow the initial oxidation products of the catechols (dopaminochrome for DA and quinone for MC) that subsequently undergo further reactions up to the formation of a melanic type precipitate.

**Figure 2:**
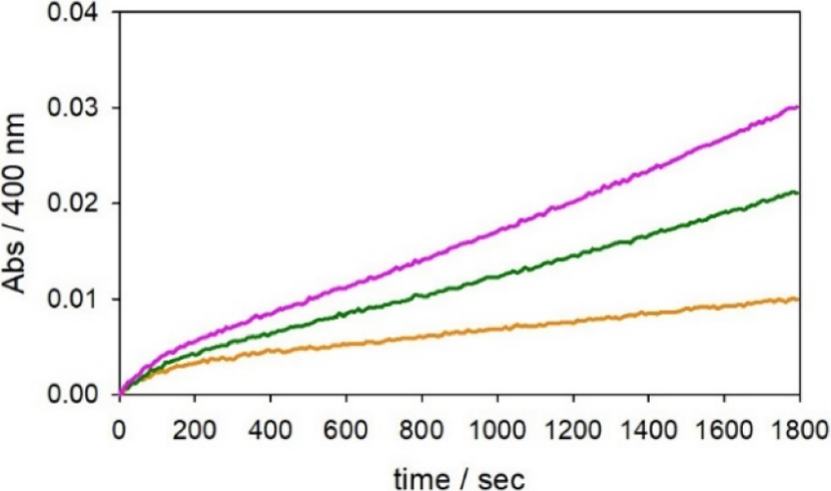
Kinetic profiles of N-acetyl DA (3 mM) oxidation with time in 50 mM HEPES buffer at pH 7.4 and 20 °C in the presence of Cu^II^ (25 μM) (orange trace) and 2 equiv NH_2_-Aβ_16_ (green) or Ac-Aβ_16_ (pink).

In fact, Cu^II^-NH_2_-Aβ_16_ appears to be more reactive than Cu^II^-Ac-Aβ_16_ toward DA but less reactive toward MC. However, DA is a competitive ligand for Cu^II^ (with a binding constant, *K*_b_, of 5 × 10^6^ M^–1^)^27^ and may compete with Ac-Aβ_16_ (*K*_b_ ∼ 10^8^ M^–1^),^28^ but not with NH_2_-Aβ_16_ (*K*_b_ ∼ 10^10^ M^–1^),^29^ at the saturating concentration; the binding constants indicate that at 3 mM DA only 22% of copper(II) is bound to Ac-Aβ_16_, while NH_2_-Aβ_16_ is able to chelate approximately 96% of free metal. The hypothesis is supported by the fact that, when N-acetyl DA (Scheme S1) was used as a substrate in the same conditions, a trend parallel to that for MC was observed (Figure 2) but also a different binding mode between DA and N-acetyl DA could be in agreement with the observed data.

To assess this point, a substrate-dependence study was performed with both MC and DA, spanning the 0.3–4.0 mM concentration range (Figure S2). For MC, the initial oxidation rates follow hyperbolic behavior. Fitting of the data with the kinetic equation reported in the Supporting Information (Scheme S2) allowed to determine the following rate constants *k*_r_ = (8.45 ± 0.3) × 10^–3^ s^–1^ and *K*_B_ (2400 ± 300) M^–1^ for Cu^II^-Ac-Aβ_16_ and *k*_r_ = (5.68 ± 0.2) × 10^–3^ s^–1^ and *K*_B_ (2200 ± 300) M^–1^ for Cu^II^-NH_2_-Aβ_16_, where *K*_B_ represents the substrate binding constant to the active species and *k*_r_ the rate constant for the oxygenation of the substrate–complex adduct (see below and Supporting Information)*.* The data confirm the higher catalytic efficiency of the former complex. In the case of DA, convergence between the initial oxidation rates was obtained for the two complexes at the saturating concentration of catecholamine. As the substrate concentration is lowered, though, the difference in rate becomes progressively larger in favor of Cu^II^-Ac-Aβ_16_. Therefore, it is clear that Cu^II^-Ac-Aβ_16_ is a more powerful oxidation catalyst than Cu^II^-NH_2_-Aβ_16_.

Even though the Cu binding mode in Cu^II^-Aβ peptides is influenced by experimental conditions such as pH, temperature, and ionic strength,^6,30−32^ blank experiments showed that catechol oxidation is not influenced by buffer. In fact, the substitution of 50 mM HEPES buffer with 50 mM phosphate buffer solution (Figure S3) as reaction *medium* does not significantly change the general trend of substrate oxidation.

According to the reaction mechanism previously proposed,^20,25,33^ the kinetic traces highlight a biphasic behavior of catechol oxidation by Cu-Aβ peptides. The initial step of the reaction leads to the generation of quinone associated with the reduction of Cu^II^-peptide to Cu^I^-peptide complex and proceeds in an oxygen independent way. To exclude the participation of dioxygen in the first seconds of the process, the reaction promoted toward sub-saturating levels of the substrate (0.3 mM DA) by Cu^II^-NH_2_-Aβ_16_ and Cu^II^-Ac-Aβ_16_ complexes (25 μM) was followed both under atmospheric oxygen and upon pre-saturation with pure dioxygen (1 atm). As suggested by the trend in Figure S4, the rate of the first phase of DA oxidation at 7.4 pH is not governed by the oxygen levels but highlights a marked dependence on the Cu complex. The faster reaction of Cu^II^-Ac-Aβ_16_ with respect to Cu^II^-NH_2_-Aβ_16_ in this initial phase is in agreement with its higher redox potential (*E*°′ +0.277 *vs* +0.178 mV).^28^

Conversely, in the second phase, increasing [O_2_] causes a rate enhancement, confirming that the reaction of dioxygen to the Cu^I^-peptide complex also contributes to the limiting step of the reaction. The substrate saturation behavior observed in the MC oxidation indicates that the coordination of catechol to the Cu^I^-peptide species is required for efficient reaction with molecular oxygen.

**Figure 3:**
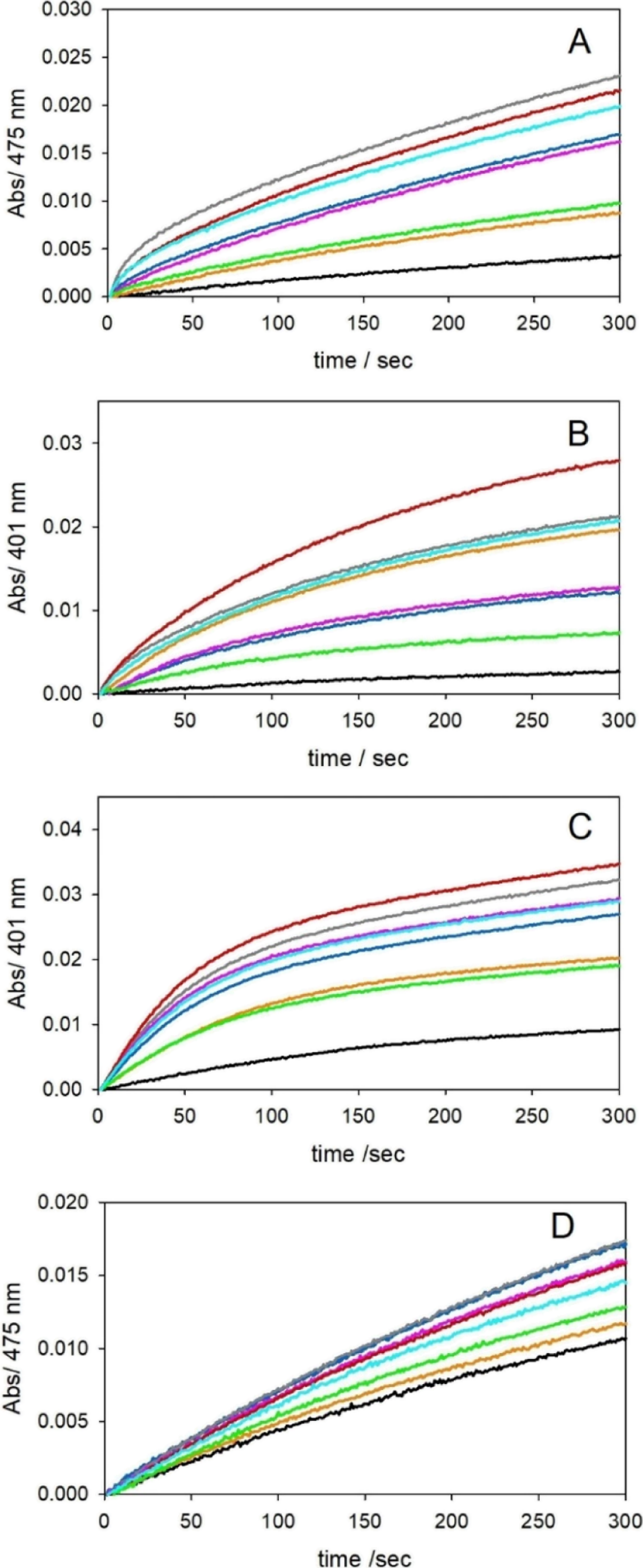
Kinetic profiles of DA (0.3 mM, panel A and 3 mM, panel D) and MC (0.3 mM, panel B and 3 mM, panel C) oxidation with time in 50 mM HEPES buffer at pH 7.4 and 20 °C; the reaction is promoted by copper(II) (25 μM) alone (orange trace), and the following complexes (25 μM): Cu^II^-NH_2_-Aβ_16_ (blue), Cu^II^-Ac-Aβ_16_ (grey), Cu^II^-NH_2_-Aβ_16_ [H6A] (pink), Cu^II^-Ac-Aβ_16_ [H6A] (red), Cu^II^-NH_2_-Aβ_16_ [H13A] (light green), and Cu^II^-Ac-Aβ_16_ [H13A] (light blue). The autoxidation of DA is shown as a black trace.

Therefore, the differences in reactivity are associated with the copper(II) and, especially, copper(I) coordination modes but it has to be considered that the substrate has an active role. To obtain more information about the Cu^I^-intermediate coordination, the isoforms of Aβ_16_ with [H6A] and [H13A] point mutations in unprotected and N-acetylated forms were synthesized. The oxidation of DA and MC both at sub-saturating (0.3 mM) and saturating concentrations (3 mM) promoted by Cu^II^-Aβ_16_[H6A], Cu^II^-Ac-Aβ_16_[H6A], Cu^II^-Aβ_16_[H13A], and Cu^II^-Ac-Aβ_16_[H13A] (25 μM) were compared with those observed with Cu^II^-NH_2_-Aβ_16_ and Cu^II^-Ac-Aβ_16_ (Figures 3 and S5–S8).

The MC oxidation was also monitored by high-performance liquid chromatography (HPLC) (Table 1 and Figure S9), quantifying the consumption of the substrate with time. The MC oxidation products obtained by HPLC separation were collected and analyzed by electrospray ionization-mass spectrometry (ESI-MS) and, when possible, by ^1^H NMR. The heterogeneity of these products (Table S1) even after a few minutes of reaction time justifies the preferred use of a simple catechol as MC with respect to DA and the need to follow the initial phase of the reaction to minimize the presence of oligomers and precipitate.

**Table 1:** HPLC Quantification of Consumed MC (0.3 mM) Obtained from Oxidation by Copper Alone (25 μM), Cu^II^-NH_2_-Aβ_16_, Cu^II^-Ac-Aβ_16_, Cu^II^-NH_2_-Aβ_16_[H13A], Cu^II^-Ac-Aβ_16_[H13A], Cu^II^-NH_2_-Aβ_16_[H6A], and Cu^II^-Ac-Aβ_16_[H6A] Complexes (25 μM, 1:1) in 50 mM HEPES Buffer at pH 7.4 and 25 °C

reaction time (min)	Cu alone (%)	[Cu-Aβ_16_] (1:1) (%)	[Cu-Ac-Aβ_16_] (1:1) (%)	[Cu-Aβ_16_(H6A)] (1:1) (%)	[Cu-Ac-Aβ_16_(H6A)] (1:1) (%)	[Cu-Aβ_16_(H13A)] (1:1) (%)	[Cu-Ac-Aβ_16_(H13A)] (1:1) (%)
5 min	14	17	21	12	27	5	20
30 min	40	29	47	26	54	14	49

The spectrophotometric and chromatographic data show a moderate but catalytic activity of the complexes. They are in agreement with the higher efficiency of the Cu complexes with N-acetylated peptides in the two phases of the reaction. The lack of the N-terminal amine decreases the stability of Cu^II^ and promotes its reduction, enhancing the initial rate. Interestingly, the data in the second rate determining phase involving dioxygen binding to the Cu^I^ species shows the highest reactivity for Cu^II^-Ac-Aβ_16_[H6A], followed by Cu^II^-Ac-Aβ_16_[H13A] and Cu^II^-Ac-Aβ_16_ (Figures 3 and S5–S8). This suggests that the coordination of only two histidine residues, together with the bound substrate, is sufficient to activate the copper(I) species. Moreover, the substitution H13A strongly affects the substrate oxidation rate, while a minor impact on the full process is observed when His6 is mutated (Figures 3 and S5–S8). These results suggest that the preferential catalytic intermediate can be generated *via* the two vicinal histidines 13 and 14, and His6 may provide an accessory binding site. It cannot be completely excluded that a transient species with Cu-His_3_ coordination contributes to the reactivity of full-length amyloid-β peptides. The low reactivity to O_2_ of two-coordinated Cu^I^-bis(imidazole) accounts for the requirement of substrate binding to enhance the reactivity of the complexes.

It is apparent that N-acetylation of Aβ removes the strong Cu^II^ binding site at the N-terminal, facilitating its reduction, and enhances the catalytic potential of the complex, requiring a minimal reorganization for Cu^II^/Cu^I^ cycling at the histidine-rich portion of the peptide. The lower catechol oxidase activity observed for Cu^II^-NH_2_-Aβ_16_ than for Cu^II^-Ac-Aβ_16_ likely depends on the competition between different binding sites for Cu^II^, which traps part of the metal into the low activity N-terminal site.

**Figure 4:**
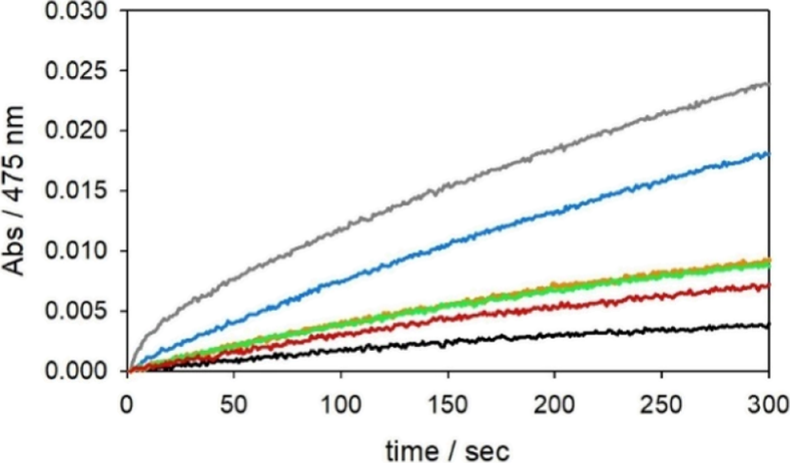
Kinetic profiles of DA (0.3 mM) oxidation with time in 50 mM HEPES buffer at pH 7.4 and 20 °C in the presence of copper(II) (25 μM) alone (orange trace), and the following complexes (25 μM): Cu^II^-NH_2_-Aβ_16_ (blue), Cu^II^-Ac-Aβ_16_ (grey), Cu^II^-Ac-Aβ_9_ (light green), and Cu^II^-NH_2_-Aβ_9_ (red). The autoxidation of DA is shown as a black trace.

To assess the catalytic potential of the copper species confined to the N-terminal Aβ portion, we investigated the reactivity of the Cu^II^ complex with the smaller peptide Aβ_9_, excluding His13 and His14.^24^ The oxidative reactivity of Cu^II^-NH_2_-Aβ_9_ and Cu^II^-Ac-Aβ_9_ toward DA was compared to the activities of the complexes with Aβ_16_ peptides (Figures 4 and S10).

As it can be seen, the oxidase activity of the two Cu complexes with NH_2_-terminal peptides is different, with Cu^II^-NH_2_-Aβ_9_ markedly less reactive than Cu^II^-Ac-Aβ_16_, and Cu^II^-NH_2_-Aβ_16_ intermediate between the two. This indicates that the enhanced redox-cycling rate of copper bound to Ac-Aβ_16_ is not because of a reorganization of the N-terminal site but to shifting of the metal binding to a more efficient bis-His intermediate that is viable for Cu^II^-NH_2_-Aβ_16_ but precluded for Cu^II^-NH_2_-Aβ_9_.

The reaction data for the catechol oxidation by the copper(II)-Aβ peptides here observed indicate that the rate determining step of the mechanism is the reaction with molecular oxygen which occurs after substrate binding to the copper(I) complex (panel A in Figures S2 and S4 and Scheme S2). The ternary [Cu^I^-peptide/catechol/O_2_] species is a key intermediate of the reaction, but it forms as a transient species that is not accumulated in solution because its reaction is faster than its formation, hindering its spectroscopic characterization. With the aim of increasing the lifetime of the [Cu^I^-peptide/catechol/O_2_] intermediate, a much less reactive catechol, that is, 4-chlorocatechol (Scheme S1), and a lower temperature were employed. In the first experiment, the complex Cu^I^-Ac-Aβ_16_ was prepared in anaerobic conditions at 6 °C and, after the addition of 0.3 mM 4-chlorocatechol, the solution was exposed to 1 atm dioxygen. Only the slow development of the bands of Cu^II^-4-chlorocatechol adduct was observed (Figure S11) without accumulation of any other transient species. A similar behavior was observed when the complex Cu^I^-Ac-Aβ_16_ was generated *in situ* by the reduction of copper(II) with 2 equiv ascorbate, followed by the addition of 4-chlorocatechol and dioxygen (Figure S12). Therefore, no features attributable to an intermediate adduct with molecular oxygen are detectable even at the lowest temperature compatible with aqueous buffer, which could be actually in agreement also with an outer sphere reaction between copper(I) and O_2_.

According to the reaction mechanism previously proposed (Scheme S2), ROS are formed during the catalytic cycle and may indeed contribute to the catechol oxidation process. In order to clarify which ROS and to what extent is formed, the effect of the presence of the scavengers dimethylsulfoxide, superoxide dismutase (SOD), and catalase on the reaction rates was investigated.^34,35^

**Figure 5:**
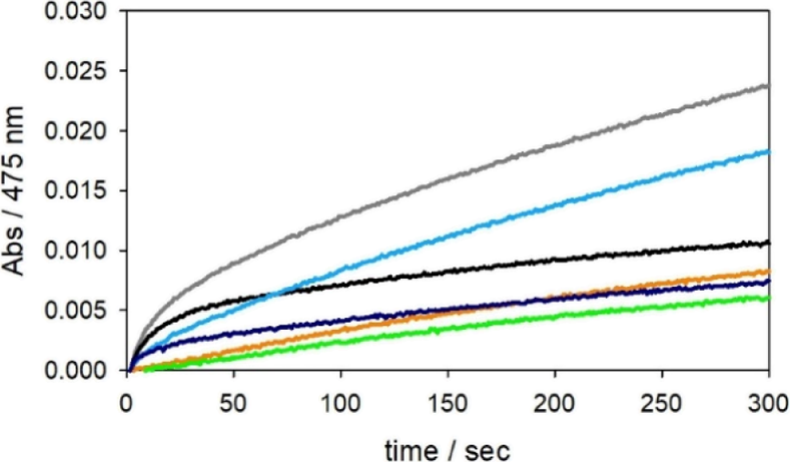
Kinetic profiles of DA (0.3 mM) oxidation with time in 50 mM HEPES buffer solution at pH 7.4 and 20 °C in the presence of Cu^II^ (25 μM) alone [orange and, upon the addition of DMSO 1.25% (v/v), shown as a green trace] and with 1 equiv NH_2_-Aβ_16_ [light blue and, upon the addition of DMSO 1.25% (v/v), blue] and 1 equiv Ac-Aβ_16_ [grey and, upon the addition of DMSO 1.25% (v/v), black].

Figure 5 shows the effect of the addition of about 1% (v/v) dimethyl sulfoxide (DMSO) in the kinetic profile for DA oxidation by the copper complexes with Ac-Aβ_16_ and NH_2_-Aβ_16_ peptides. The absorbance changes with time are reduced, indicating slower DA oxidation. It should be noted that the effect of DMSO could be attributed only partly to its hydroxyl radical scavenger effect because also its affinity toward copper(I) will slow down the reaction with dioxygen.

**Figure 6:**
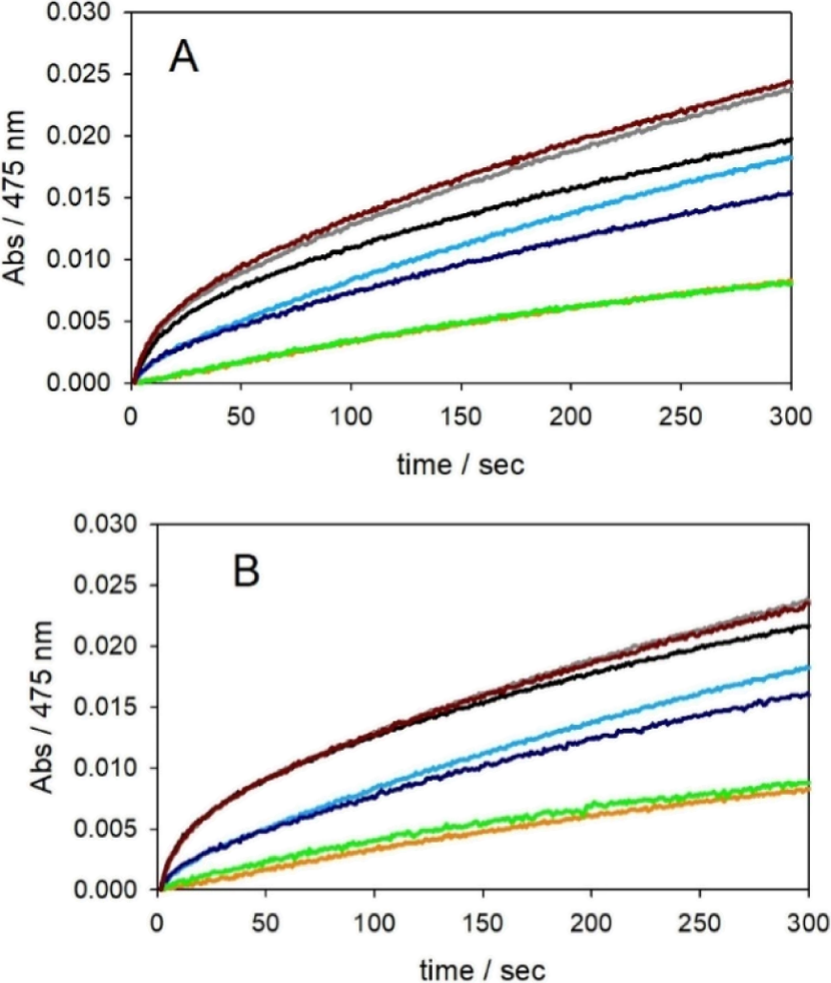
Kinetic profiles of DA (0.3 mM) oxidation with time in 50 mM HEPES buffer solution at pH 7.4 and 20 °C in the presence of Cu^II^ (25 μM) alone [orange and, upon the addition of SOD enzyme in panel A or catalase in panel B (500 units/1.6 mL), shown as a green trace] and with 1 equiv NH_2_-Aβ_16_ (light blue and, upon the addition of each enzyme, blue) and 1 equiv Ac-Aβ_16_ (grey and, upon the addition of the active enzyme, as a black profile, or denatured enzyme, as a brown trace).

Figure 6A reports the effect of the addition of SOD, and its inactivated form, in the typical reaction conditions of the DA oxidation experiment promoted by copper(II) or copper(II)-Aβ peptides described above. Clearly, also the presence of SOD slows down DA oxidation. The effect is not because of copper removal by SOD because the denaturated enzyme has no effect (Figure 6, brown trace). These results indicate that the superoxide anion is formed during the catalytic cycle and contributes to DA oxidation, albeit to a modest extent.

Similarly, Figure 6B reports that the addition of catalase or the inactivated enzyme in the reaction *medium* reduces the DA oxidation rate, again to a minor extent. This behavior indicates that hydrogen peroxide is a ROS species that is formed during the reaction and gives some contribution to DA oxidation.

**Figure 7:**
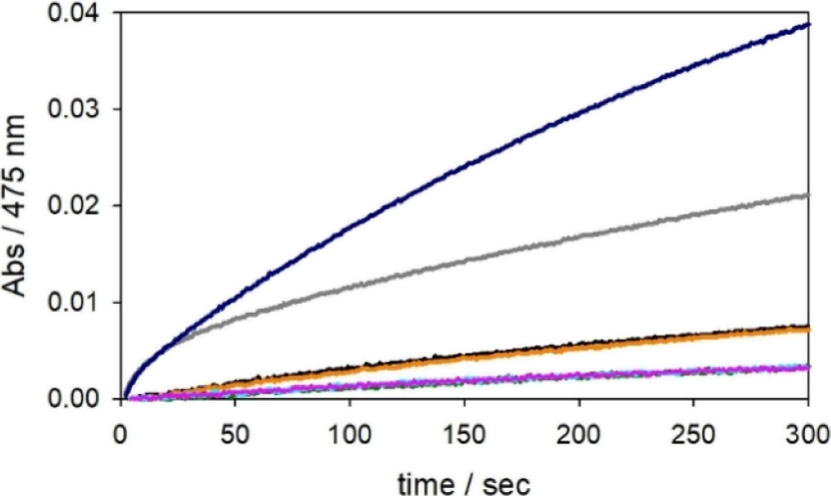
Kinetic profiles of DA (0.3 mM) oxidation with time in 50 mM HEPES buffer solution at pH 7.4 and 20 °C in the presence of Cu^II^ (25 μM) alone (orange trace) and H_2_O_2_ (0.25 mM, black). The reaction traces for DA autoxidation, DA oxidation by H_2_O_2_ alone, or upon the addition of 1 equiv Ac-Aβ_16_ (25 μM), completely overlap and are shown as green, light blue, or pink traces, respectively. The reactions promoted by Cu^II^-Ac-Aβ_16_ at a 1:1 M ratio (25 μM) in the absence and presence of H_2_O_2_ (0.25 mM) are shown as grey and blue profiles.

To further assess the effect of H_2_O_2_ in the reaction, the oxidation of DA by the copper complexes was studied adding a large excess of hydrogen peroxide with respect to Cu (Figure 7). The increase in DA oxidation with time shows that H_2_O_2_ contributes to the reaction because it reacts with copper forming species that are significantly reactive. Interestingly, hydrogen peroxide has almost no effect in the first seconds of the reaction, involving catechol oxidation by copper(II), while it increases the rate of the second phase, suggesting it reacts with copper(I), perhaps by oxidizing the metal ion to the more reactive +2 form and/or forming an additional reactive Cu^II^-hydroperoxo intermediate.

### Biological Relevance and Conclusions

The present study shows that the rate of catechol oxidase reaction by Cu^II^-Aβ depends on both dioxygen and catechol concentrations. The coordination of N-terminal amine reduces the efficiency of Cu^II^-peptide complexes in the reaction, stabilizing the Cu^II^ state and requiring a structural rearrangement upon metal reduction. Indeed, the *N*-terminally confined intermediate is a poor catalyst of catechol oxidation because the coordination set comprising the hard O(carboxylate) and NH_2_ ligands makes the Cu^I^ species unsuitable for efficient binding and activation of dioxygen.^36^

**Scheme 1:**
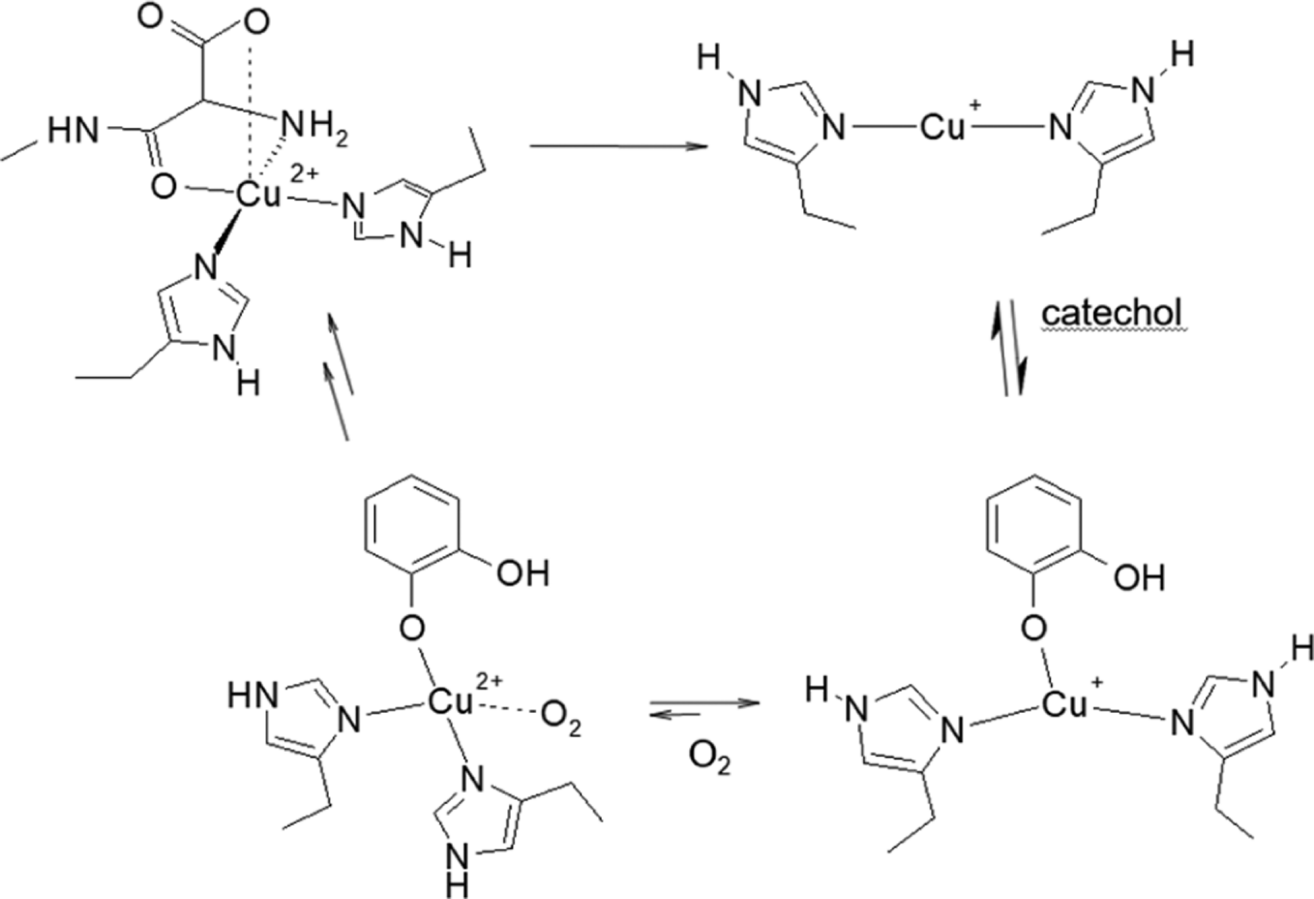
Representation of the Productive Pathway of Cu^II^-Aβ Activation in the Oxidase Reaction Reduction of Cu^II^-Aβ by a molecule of catechol leads to the formation of the Cu^I^-bis(histidine) species that becomes reactive to O_2_ upon catechol binding and promotes the oxidase reaction.

When the N-terminus is protected, Cu^II^ is directed to the preferential Aβ_16_ C-terminal site, where reduction to Cu^I^ becomes fast and a Cu^I^-bis(imidazole) species involving the His-tandem is highly stabilized. Reoxidation of this complex requires the binding of the external substrate, warping the linear coordination and promoting O_2_ binding (Scheme 1). The nonphysiological *N*-protected form, Ac-Aβ_16_, enables to increase the relative amount of Cu^II^-bis(imidazole) species initially present at equilibrium, strengthening the catecholase activity of the complex.

Interestingly, in the catalytic oxidation of ascorbate, the order of reactivity of Cu^II^ complexes with Ac-Aβ_16_ and NH_2_-Aβ_16_ peptides is opposite to that found here in the oxidation of catechols.^17,28,37^ In that case, a “catalytic in-between state” with the previously described coordination mode and possibly a bound substrate has been proposed. The different coordination modes of the reactive species exhibited in ascorbate or catechol oxidation could be related to the different one-electron reduction potential of the two substrates (lower for ascorbate), to the intrinsic copper catalytic activity (higher with ascorbate), and to the substrate coordination effect in the Cu^I^ intermediate. In the catechol oxidation, the rate is ruled by the formation of the ternary complex of Cu^I^/O_2_/catechol,^25^ which is stabilized with the 2His-1catechol coordination set allowed by Ac-Aβ_16_, and makes possible the reaction with dioxygen.

During the reaction, hydrogen peroxide and superoxide are formed as side products; these reactive species do not simply accumulate in solution but also participate in DA oxidation, in a minor but a nonnegligible extent. More generally, this study provides further details about the reactivity of copper bound to β-amyloid peptides that are a prerequisite for a better understanding of the biochemical mechanisms leading to oxidative stress and to the exacerbation of AD.

## Experimental Section

### Materials and Instrumentation

Protected amino acids, rink amide resin, and other reagents for peptide synthesis were purchased from Novabiochem. All other chemicals were of reagent grade from Sigma-Aldrich. Peptide purifications were performed on a Shimadzu HPLC instrument equipped with two LC-20AD pumps and a SPD-M20A diode array detector (working range: 190–800 nm), using a Phenomenex Jupiter 4U Proteo semipreparative column (4 μm, 250 × 10 mm). UV–vis spectra and kinetic experiments were recorded on an Agilent 8453 diode array spectrophotometer, equipped with a thermostated, magnetically stirred optical cell.

### Peptide Synthesis

The eight peptides NH_2_-Aβ_16_ (NH_2_-D_1_AEFRHDSGYEVHHNK_16_), Ac-Aβ_16_ (Ac-D_1_AEFRHDSGYEVHHNK_16_), NH_2_-Aβ_16_[H6A] (NH_2_-D_1_AEFRADSGYEVHHNK_16_), Ac-Aβ_16_[H6A] (Ac-D_1_AEFRADSGYEVHHNK_16_), NH_2_-Aβ_16_[H13A] (NH_2_-D_1_AEFRHDSGYEVAHNK_16_), Ac-Aβ_16_[H13A] (Ac-D_1_AEFRHDSGYEVAHNK_16_), NH_2_-Aβ_9_ (NH_2_-D_1_AEFRHDSG_9_), and Ac-Aβ_9_ (Ac-D_1_AEFRHDSG_9_) were synthesized using the standard fluorenyl methoxycarbonyl (Fmoc) solid-phase synthesis in dimethylformamide (DMF).^38,39^ Rink-amide resin MBHA (substitution 0.58 mmol/g) was used as the polymeric support, which yielded the peptide amidated at the C-terminus. The deprotection of the resin and of the Fmoc group from each amino acid was performed with 20 mL of 20% (v/v) piperidine in DMF, repeating the reaction twice, for 3 and 7 min. Each amino acid (2 mol equiv *vs* resin sites) was added in the presence of 2 equiv of *N*-hydroxybenzotriazole, 2 equiv of benzotriazol-1-yloxytripyrrolidinophosphonium hexafluorophosphate, and ∼2 equiv of *N,N*-diisopropyl ethylamine. The coupling reaction proceeds for at least 45 min. After recoupling of each amino acid, a capping step was performed by using 20 mL of 4.7% acetic anhydride and 4% of pyridine in DMF; the resin was washed with DMF, dichloromethane, and isopropanol. At the end of the synthesis, the protections of the side chains of the amino acids were removed with a solution of 95% trifluoroacetic acid (TFA, 25 mL for 1 g of resin), triisopropyl silane (2.5%), and water (2.5%). After stirring for 3 h, cold diethyl ether was added to precipitate the peptide and the mixture was filtered; then, it was dissolved in water and purified by HPLC, using a 0–100% linear gradient of 0.1% TFA in water to 0.1% TFA in CH_3_CN over 50 min (flow rate of 4 mL/min, loop 2 mL) as the eluent. The identity of the peptides was confirmed by ESI-MS (Thermo-Finnigan). ESI-MS data (direct injection, MeOH, positive-ion mode, capillary temperature 200 °C): *m*/*z* 1955 (Aβ_16_H)^+^; 978 (Aβ_16_H_2_)^2+^; 652 (Aβ_16_H_3_)^3+^; 489 (Aβ_16_H_4_)^4+^; 1997 (Ac-Aβ_16_H)^+^; 999 (Ac-Aβ_16_H_2_)^2+^; 666 (Ac-Aβ_16_H_3_)^3+^; 500 (Ac-Aβ_16_H_4_)^4+^; 1033 (Aβ_9_H)^+^; 517 (Aβ_9_H_2_)^2+^; 345 (Aβ_9_H_3_)^3+^; 1075 (Ac-Aβ_9_H)^+^; 538 (Ac-Aβ_9_H_2_)^2+^; 359 (Ac-Aβ_9_H_3_)^3+^; 1889 (Aβ_16_[H6A]H and Aβ_16_[H13A]H)^+^; 945 (Aβ_16_[H6A]H_2_ and Aβ_16_[H13A]H_2_)^2+^; 630 (Aβ_16_[H6A]H_3_ and Aβ_16_[H13A]H_3_)^3+^; 473 (Aβ_16_[H6A]H_4_ and Aβ_16_[H13A]H _4_)^4+^; 1931 (Ac-Aβ_16_[H6A]H and Ac-Aβ_16_[H13A]H)^+^; 966 (Ac-Aβ_16_[H6A]H_2_ and Ac-Aβ_16_[H13A]H_2_)^2+^; 644 (Ac-Aβ_16_[H6A]H_3_ and Ac-Aβ_16_[H13A]H_3_)^3+^; 483.5 (Ac-Aβ_16_[H6A]H_4_ and Ac-Aβ_16_[H13A]H _4_)^4+^.

### Quantification of Peptide Solutions

Quantification of the peptide solutions for NH_2_-Aβ_16_, Ac-Aβ_16_, NH_2_-Aβ_16_[H6A], Ac-Aβ_16_[H6A], NH_2_-Aβ_16_[H13A], and Ac-Aβ_16_[H13A] was performed by UV–visible absorption at 280 nm corresponding to the tyrosine band (ε 1480 M^–1^ cm^–1^).^40^ With NH_2_-Aβ_9_ and Ac-Aβ_9_, the quantification was made by weighing the dry peptides.

### Oxidation Kinetics at Saturating Concentration of the Substrate

The catalytic oxidation of the DA, MC, and N-acetyl DA by Cu^II^ was studied at 20 °C for 1800 s in 50 mM HEPES buffer at pH 7.4. The synthesis of *N*-acetyl DA was performed as described above.^41^ The reaction was monitored by UV–visible spectroscopy through the development of the dopaminochrome band at 475 nm for DA and the quinone band at around 400 nm for MC and N-acetyl DA. The substrate (3 mM) autoxidation reaction was also evaluated. All experiments were carried out by adding copper(II) nitrate (25 μM) to the substrate (3 mM) and 1 or 2 equiv of NH_2_-Aβ_16_ and Ac-Aβ_16_ peptides (25–50 μM). The same conditions were maintained for the catalytic studies performed in the presence of copper(II) bound to the mutant peptides but fixing the complex concentration at 25 μM. All measurements were performed at least in duplicate.

### Oxidation Kinetics at Low Concentrations of the Substrate

The catechol oxidation promoted by Cu^II^ was studied at 20 °C for 300 s in 50 mM HEPES buffer at pH 7.4 and in 50 mM phosphate buffer solution at pH 7.4 to exclude the buffer involvement in the reaction mechanism. The reactions were followed as described above. The substrate (0.3 mM) autoxidation experiment was also evaluated. All experiments were carried out by adding copper(II) nitrate (25 μM) and amyloid-β fragments at a 1:1 M ratio to the substrate solution (0.3 mM). In order to assay the participation of ROS in the oxidative reactions promoted by copper-peptide complexes, a comparative study was performed in which the reaction toward the substrate (0.3 mM DA) was monitored both in the previous conditions and upon the addition of the scavengers DMSO (1.25% v/v), SOD and catalase (500 units/1.6 mL). These enzymes were also used in the denaturated form by heating their solutions at 100 °C for 1 h. The involvement of hydrogen peroxide in the DA oxidation reaction was also investigated by the addition of H_2_O_2_ (0.25 mM), with both Cu^II^ alone or Cu^II^-Ac-Aβ_16_ maintaining the previously described conditions. In the attempt to trap the Cu intermediate of the reaction, the reduced complexes Cu^I^-Ac-Aβ_16_ (25 μM) were generated in anaerobic conditions at 6 °C, either by directly adding tetrakis(acetonitrile)copper(I) hexafluorophosphate (25 μM) or generating Cu^I^ from copper(II) nitrate and 2 equiv ascorbate (50 μM). Once the mixture had been saturated with argon and the spectra acquisition started, 4-chlorocatechol (0.3 mM) was added to the solution and the mixture was then exposed to 1 atm dioxygen. All measurements were performed at least in duplicate.

### Substrate-Dependence Kinetics

The kinetic data were obtained performing the oxidation of DA (followed at 475 nm, ε = 3300 M^–1^ cm^–1^)^42^ and MC (followed at 401 nm, ε = 1550 M^–1^ cm^–1^)^43^ in the presence of a fixed concentration of the copper-peptide complex (at 1:1 M ratio, 25 μM). The substrate dependence was investigated by varying the catechol concentration from 0.3 to 4.0 mM. The rate values were converted from ΔAbs/s into s^–1^ (rate/[catalyst]) through the Beer equation. The metal ion contributions to the reaction rates were obtained subtracting the substrate autoxidation contribution. The initial rates obtained for MC were fitted with eq S1 (see Scheme S2, Supporting Information).

### HPLC Quantification of MC Consumption

The oxidation of MC in 50 mM HEPES buffer at pH 7.4 and 25 °C was evaluated through HPLC, using a 0–50% linear gradient of 0.1% TFA in water to 0.1% TFA in CH_3_CN over 40 min (flow rate of 4 mL/min, loop 2 mL) as the eluent. For each sample, a stock solution of MC (0.3 mM) was previously prepared and divided into three aliquots analyzed at the initial conditions (zero time), after 5 and 30 min. The reaction started with the addition of copper(II) nitrate (25 μM) to the solution of the substrate and of each peptide fragments, NH_2_-Aβ_16_, Ac-Aβ_16_, NH_2_-Aβ_16_[H6A], Ac-Aβ_16_[H6A], NH_2_-Aβ_16_[H13A], and Ac-Aβ_16_[H13A] (25 μM). In order to quantify the consumption of MC, an internal standard (kojic acid, 0.1 mM) was added to the solutions just before injection. Peaks corresponding to the oxidation products were collected and the solutions rotary evaporated to characterize the mixture composition; the identification of oxidation compounds was performed through ESI-MS and, when allowed by the amount isolated, through ^1^H NMR (data not shown).

## Supporting Information Available

UV–visible spectra, kinetic plots, HPLC chromatograms, and qualitative characterization of the oxidative products (PDF)
