# Supplementary material for: A Cu-bis(imidazole) Substrate Intermediate Is the Catalytically Competent Center for Catechol Oxidase Activity of Copper Amyloid-β
Source: Inorg Chem. 2021 Jan 6;60(2):606–13. doi: 10.1021/acs.inorgchem.0c02243 (PMC8023651; doi:10.1021/acs.inorgchem.0c02243)
Supplement: Supplementary file 1 — ic0c02243_si_001.pdf [file ic0c02243_si_001.pdf]

Supporting Information

A Cu-bis(imidazole)-substrate intermediate is the catalytically competent center for catechol oxidase activity of copper-amyloid-β

Chiara Bacchella, Simone Dell'Acqua, Stefania Nicolis, Enrico Monzani,* Luigi Casella*

Dipartimento di Chimica, Università di Pavia, Via Taramelli 12, 27100 Pavia. Italy

AUTHOR INFORMATION

Corresponding Authors: *enrico.monzani@unipv.it; * luigi.casella@unipv.it

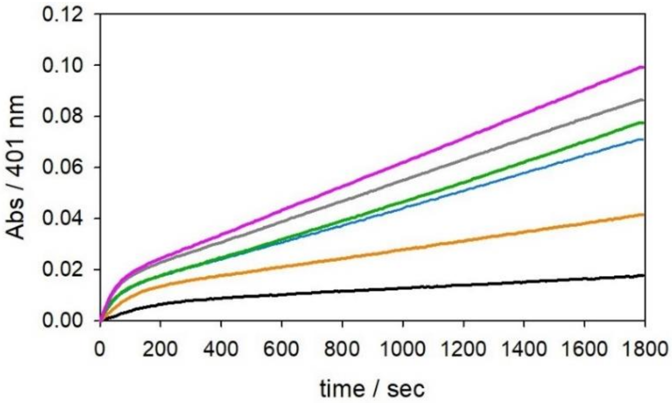

Figure S1. Kinetic profiles of MC (3 mM) oxidation with time in 50 mM HEPES buffer solution at pH 7.4 and 20 °C (autoxidation, black trace) in presence of Cu^{II} (25 μM) (orange) and with 1 equiv. NH_2-Aβ_{16} (blue), 2 equiv. NH_2-Aβ_{16} (green), and 1 equiv. Ac-Aβ_{16} (grey) and 2 equiv. Ac-Aβ_{16} (pink).

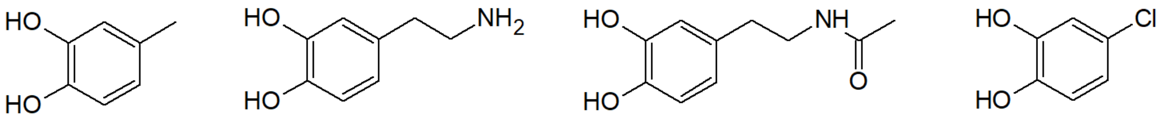

1 2 3 4

Scheme S1. Chemical structures of 4-methylcatechol (1), dopamine (2), *N*-acetyl-dopamine (3) and 4-chlorocatechol (4).

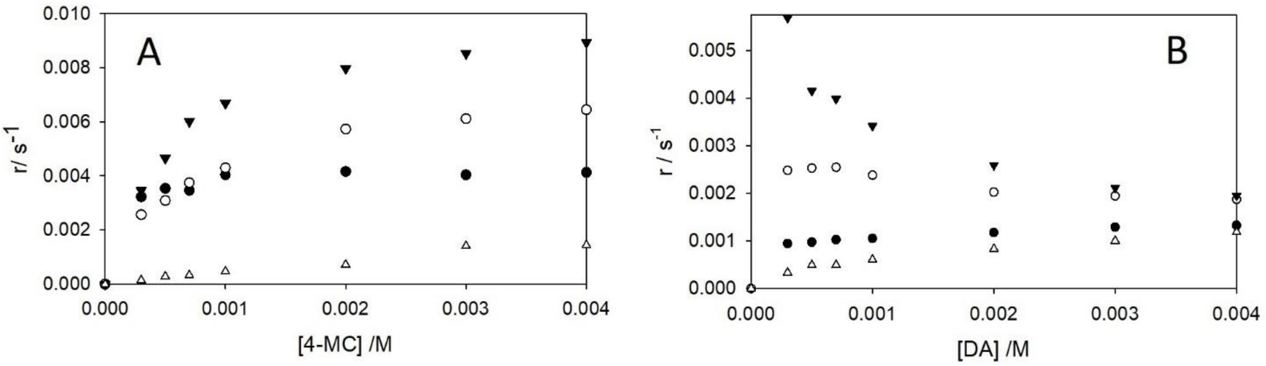

Figure S2. Dependence on [MC] (panel A) and [DA] (panel B) of the initial oxidation rates (indicated as r = rates/[catalyst]) in the presence of catalyst (Cu or Cu-peptide complex in 1:1 molar ratio, 25 μM). The concentration of substrate varies from 0.3 to 4.0 mM and its oxidation was studied in only HEPES buffer (white triangles) and in the presence of Cu^{II} alone (black circles), Cu^{II}-NH_2-$A\beta_{16}$ (white circles) and Cu^{II}-Ac-$A\beta_{16}$ (black triangles). The data for catechol autoxidation (white triangles) were obtained assuming a copper concentration of 25 μM.

1) Cu^{II}-Aβ + MC ⟶ Cu^{I}-Aβ + $MC^{+\bullet}$ *Fast Reaction*
2) Cu^{I}-Aβ + MC ⇄ Cu^{I}-Aβ-MC *Fast Equilibrium*
3) Cu^{I}-Aβ-MC + O_2 ⟶Cu-Aβ-MC-O_2* *Slow Reaction*
4) Cu-Aβ-MC-O_2* ⟶⟶Cu^{II}-Aβ + $MC^{+\bullet}$ + ROS *Fast Reactions*
5) 2 $MC^{+\bullet}$ ⟶ MC + MQ *Chromophore Formation*
6) MC + MQ ⟶⟶ oligomers *Oligomerization*

Equation S1) $$\frac{\text{rate}}{[Cu^{II}\text{-}A\beta]} = \frac{k_r \times K_B \times [MC]}{1 + K_B \times [MC]}$$

Scheme S2. Simplified mechanism showing the main steps followed by Cu-peptide complex in the catalytic oxidation of catechols, followed by the equation used for the fitting of substrate-dependence data. Aβ indicates NH_2-$A\beta_{16}$ or Ac-$A\beta_{16}$ peptides, $MC^{+\bullet}$ indicates the radical cation of MC, K_B is the binding constant for equilibrium 2, i.e. the substrate binding to the copper(I)-peptide complex, and k_r is the second order rate constant for dioxygen binding to the Cu^{I}-Aβ-MC species. In deriving the kinetic equation it was considered that oxygenation of Cu^{I}-Aβ (with no substrate bound) could be neglected (as experimentally observed). Cu-Aβ-MC-O_2* indicates generically the active species formed that cannot be spectroscopically characterized due to its fast reaction/decomposition.

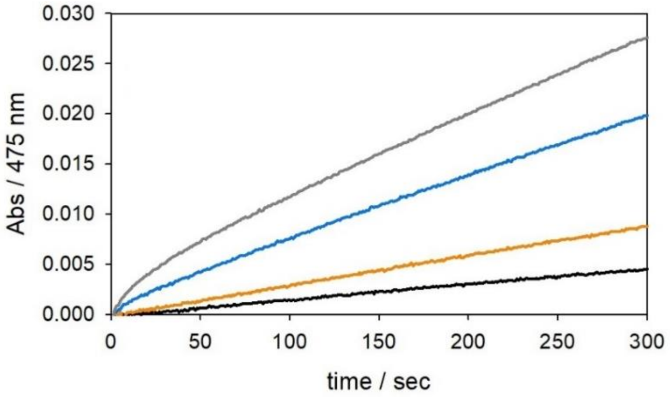

Figure S3. Kinetic profiles of DA (0.3 mM) oxidation with time in phosphate buffer solution (50 mM) at pH 7.4 and 20 °C in the presence of copper(II) (25 μM) alone (orange trace), and the following complexes (25 μM): Cu^{II}-NH_2-$A\beta_{16}$ (blue) and Cu^{II}-Ac-$A\beta_{16}$ (grey). The autoxidation of DA is shown as black trace.

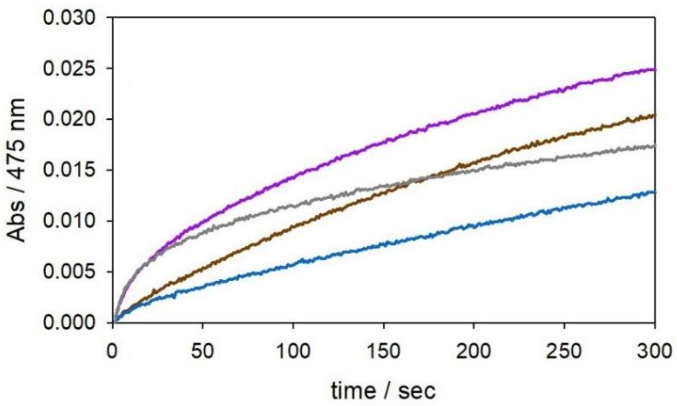

Figure S4. Kinetic profile of DA (0.3 mM) oxidation with time in 50 mM HEPES buffer solution at pH 7.4 and 20 °C in the presence of Cu^{II}-NH_2-Aβ_{16} (25 μM) (blue trace) and Cu^{II}-Ac-Aβ_{16} (grey). The same experiment was performed upon saturation of buffer solution with pure oxygen (brown, Cu^{II}-NH_2-Aβ_{16}, and violet, Cu^{II}-Ac-Aβ_{16}).

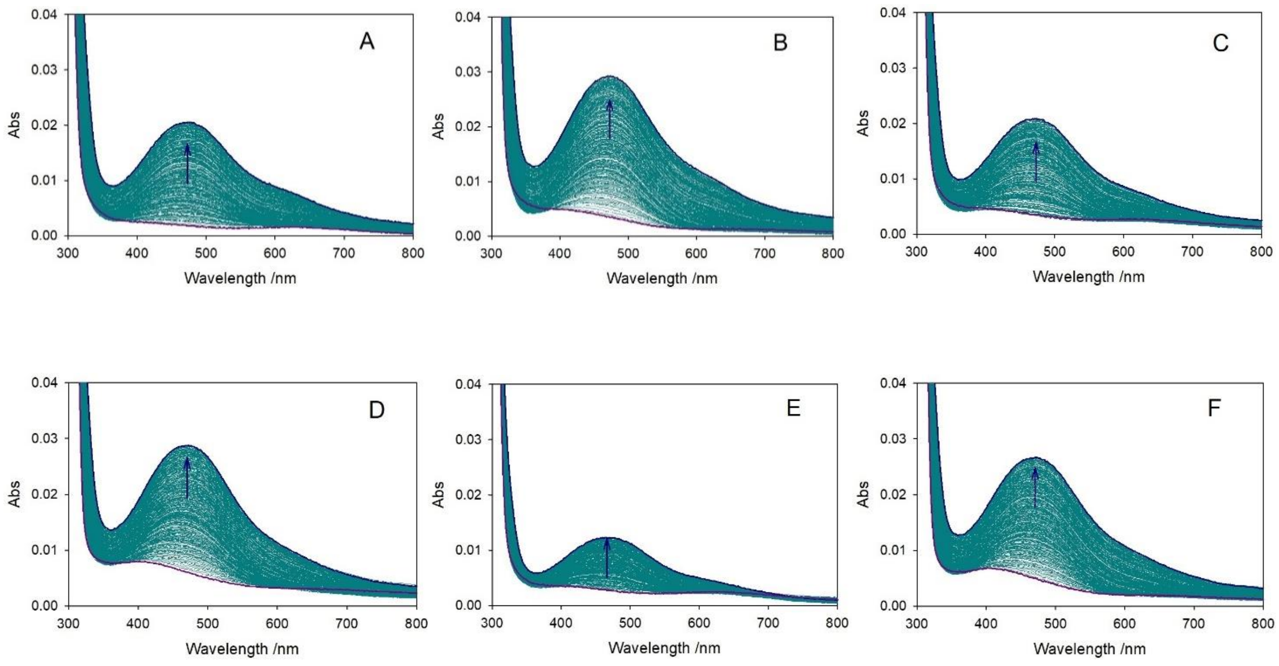

Figure S5. Absorbance changes with time during DA (0.3 mM) oxidation in HEPES buffer (50 mM) at pH 7.4 and 20 °C in the presence of the following complexes (25 μM): Cu^{II}-NH_2-Aβ_{16} (panel A), Cu^{II}-Ac-Aβ_{16} (panel B), Cu^{II}-NH_2-Aβ_{16} [H_6A] (panel C), Cu^{II}-Ac-Aβ_{16} [H_6A] (panel D), Cu^{II}-NH_2-Aβ_{16} [H_{13}A] (panel E) and Cu^{II}-Ac-Aβ_{16} [H_{13}A] (panel F).

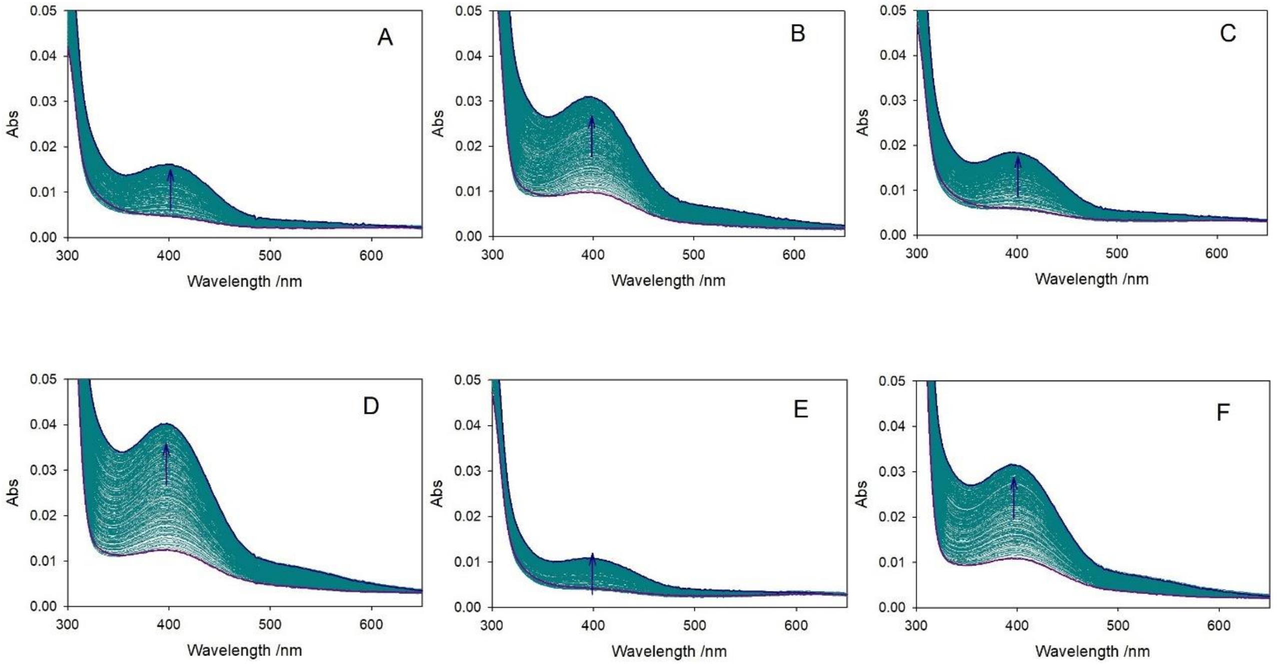

Figure S6. Absorbance changes with time during MC (0.3 mM) oxidation in HEPES buffer (50 mM) at pH 7.4 and 20 °C in the presence of the following complexes (25 μM): Cu^{II}-NH_2-$A\beta_{16}$ (panel A), Cu^{II}-Ac-$A\beta_{16}$ (panel B), Cu^{II}-NH_2-$A\beta_{16}$ $[H_6A]$ (panel C), Cu^{II}-Ac-$A\beta_{16}$ $[H_6A]$ (panel D), Cu^{II}-NH_2-$A\beta_{16}$ $[H_{13}A]$ (panel E) and Cu^{II}-Ac-$A\beta_{16}$ $[H_{13}A]$ (panel F).

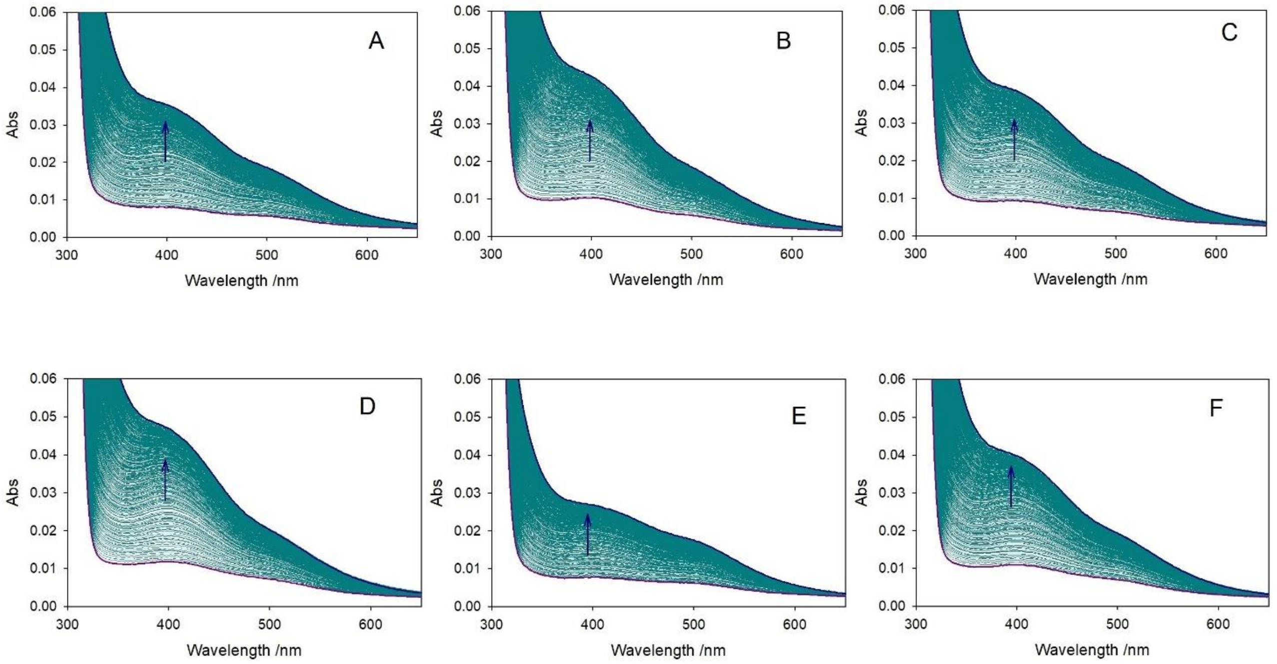

Figure S7. Absorbance changes with time during MC (3 mM) oxidation in HEPES buffer (50 mM) at pH 7.4 and 20 °C in the presence of the following complexes (25 μM): Cu^{II}-NH_2-$A\beta_{16}$ (panel A), Cu^{II}-Ac-$A\beta_{16}$ (panel B), Cu^{II}-NH_2-$A\beta_{16}$ $[H_6A]$ (panel C), Cu^{II}-Ac-$A\beta_{16}$ $[H_6A]$ (panel D), Cu^{II}-NH_2-$A\beta_{16}$ $[H_{13}A]$ (panel E) and Cu^{II}-Ac-$A\beta_{16}$ $[H_{13}A]$ (panel F).

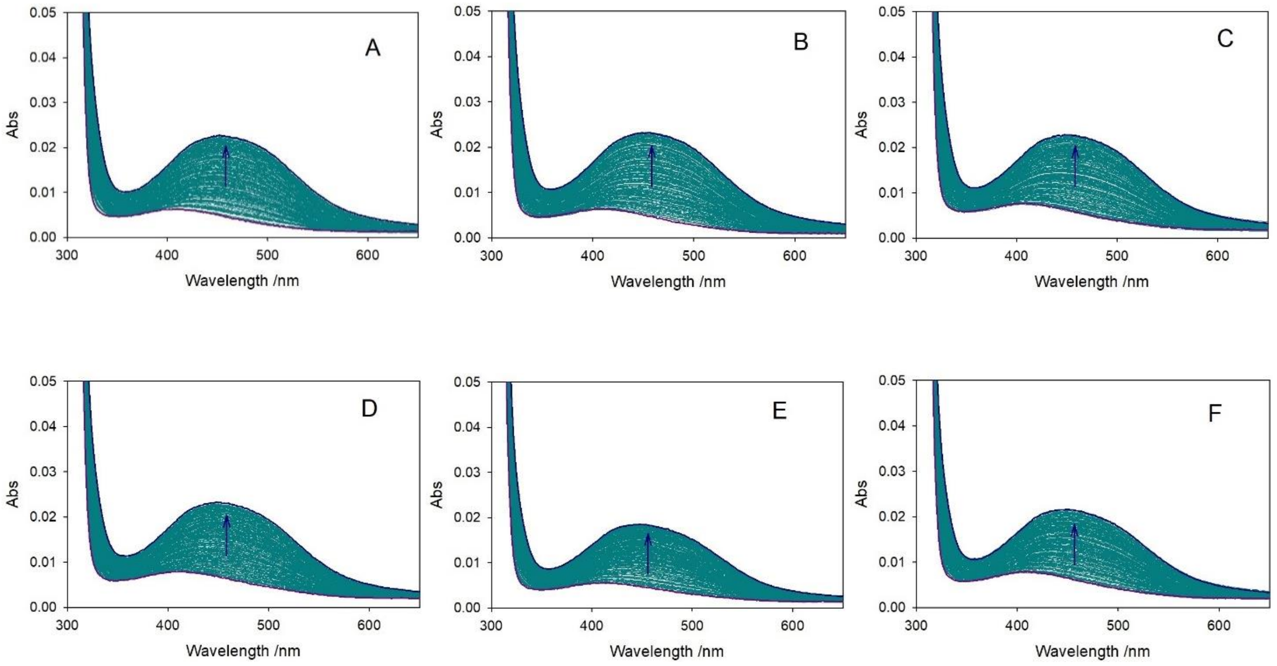

Figure S8. Absorbance changes with time during DA (3 mM) oxidation in HEPES buffer (50 mM) at pH 7.4 and 20 °C in the presence of the following complexes (25 μM): Cu^{II}-NH_2-Aβ_{16} (panel A), Cu^{II}-Ac-Aβ_{16} (panel B), Cu^{II}-NH_2-Aβ_{16} [H_6A] (panel C), Cu^{II}-Ac-Aβ_{16} [H_6A] (panel D), Cu^{II}-NH_2-Aβ_{16} [H_{13}A] (panel E) and Cu^{II}-Ac-Aβ_{16} [H_{13}A] (panel F).

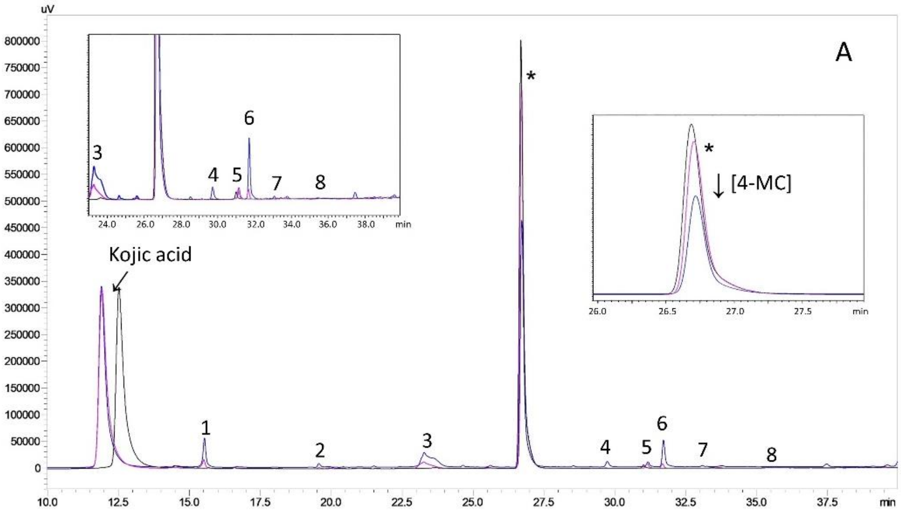
A
uV
Kojic acid
[4-MC]
min
1
2
3
4
5
6
7
8
*

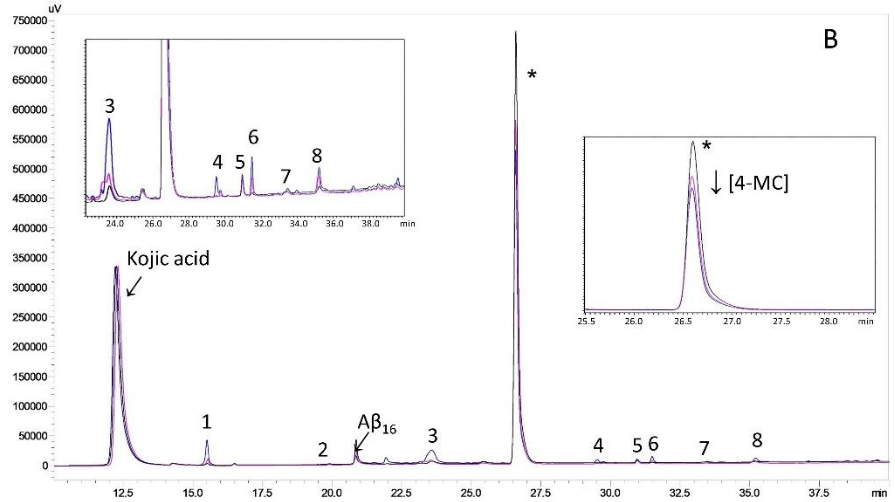
B
uV
Kojic acid
Aβ16
[4-MC]
min
1
2
3
4
5
6
7
8
*

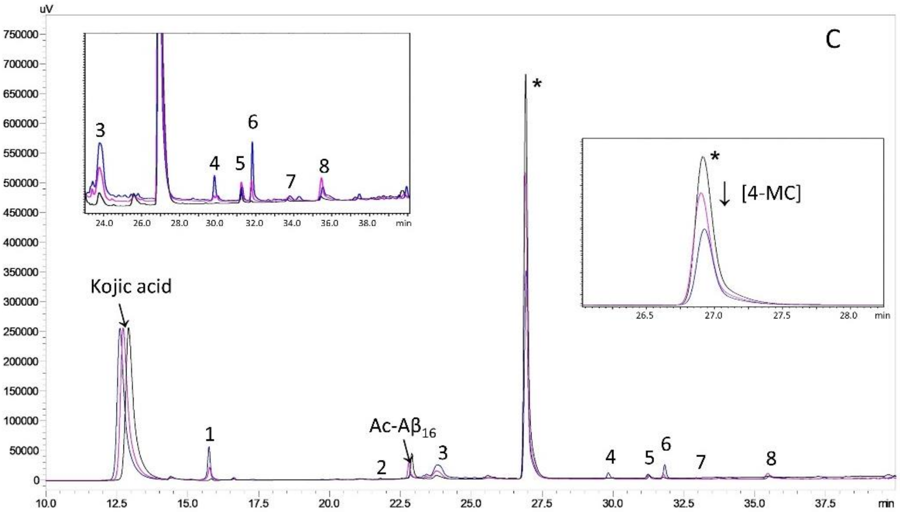
C
uV
Kojic acid
Ac-Aβ16
[4-MC]
min
1
2
3
4
5
6
7
8
*

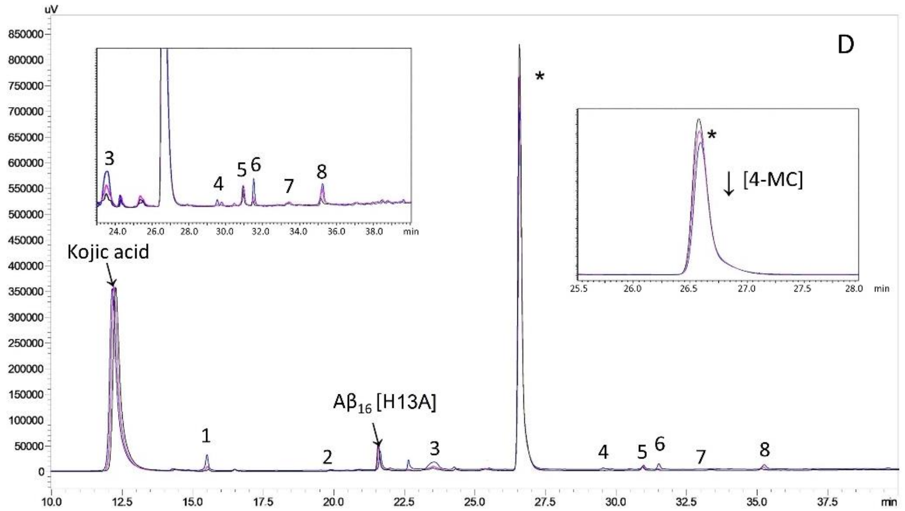
D
Kojic acid
Aβ16 [H13A]
[4-MC]
uV
min

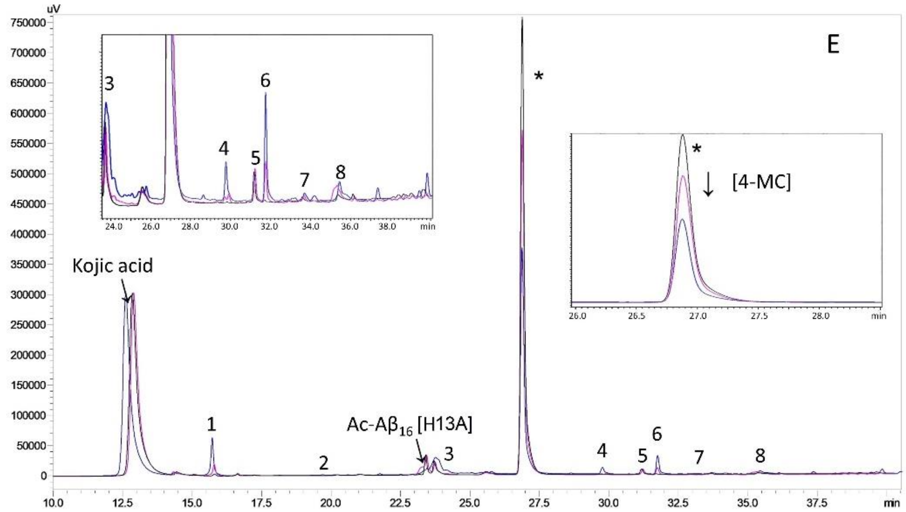
E
Kojic acid
Ac-Aβ16 [H13A]
[4-MC]
uV
min

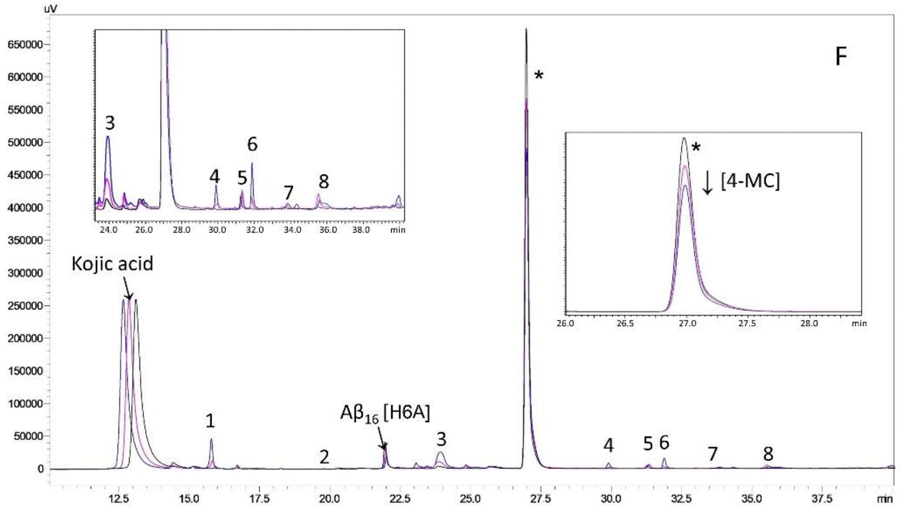
F
Kojic acid
Aβ16 [H6A]
[4-MC]
uV
min

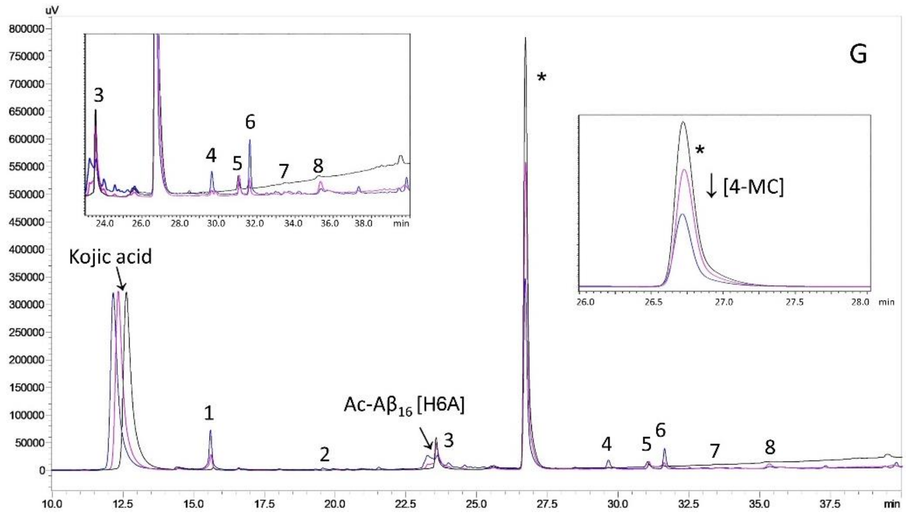

Figure S9. HPLC chromatograms showing MC consumption (0.3 mM) with time in HEPES buffer (50 mM) at pH 7.4 and 25 °C in the presence of copper(II) (25 μM) alone (panel A), and the following complexes (25 μM): Cu^{II}-NH_2-$A\beta_{16}$ (panel B), Cu^{II}-Ac-$A\beta_{16}$ (panel C), Cu^{II}-NH_2-$A\beta_{16}$ [H13A] (panel D), Cu^{II}-Ac-Aβ16[H_{13}A] (panel E), Cu^{II}-NH_2-$A\beta_{16}$ [H6A] (panel F) and Cu^{II}-Ac-$A\beta_{16}$[H6A] (panel G). The peak eluted after 12.5 minutes was used as internal standard (kojic acid, 0.1 mM) to quantify the substrate oxidation (r.t. 26.5 min). The mixture was analyzed at time points 0, 5 and 30 min, shown as black, pink and blue profiles, respectively. See Table S1 for the characterization of oxidative products.

Table S1. Qualitative characterization of the oxidative products generated by the reaction of MC with copper(II) alone or complexed with β-amyloid peptides; each peak numbered in the HPLC chromatograms was analyzed through ESI-MS and, when possible, by ^{1}H-NMR.

Peak Number	*Retention time (min)*	*ESI/MS (m/z)*	*products* *
1	16	245(+)	MC/MQ-dimer
2	19.5	311(+)	MC-dimer with 4 H_2O
		328(+)	MC-dimer with 5 H_2O
3	23.5	439(+)	MC-trimer with 3 H_2O + Na^+
		457(+)	MQ-trimer with 6 H_2O
4	29	276(+)	MC/MQ-dimer with 2 H_2O
		371(+)	MC-trimer
5	31	246(+)	MC-dimer
		262(+)	MC-dimer with 1 H_2O
6	31.7	416(+)	MC-trimer with 3 H_2O
		439(+)	MC-trimer with 3 H_2O + Na^+
7	33.5	245(+)	MC/MQ-dimer
		262(+)	MC-dimer with 1 H_2O
8	35.5	245(+)	MC/MQ-dimer

** The complexity of composition of oxidation products is due to the easy oxidation of catechols to the corresponding quinones and the susceptibility of the formed quinones to undergo nucleophilic addition and re-aromatization. When the nucleophile is water, the oxidation/nucleophilic addition reactions give rise to a final oxygen atom (+16 amu) addition with respect to the initial catechol. When the nucleophile is a catechol, an oligomerization process occurs. The compounds formed by these reactions are extensively conjugated catechols. They can be further oxidized to their corresponding quinones and then undergo nucleophilic additions in a chain reaction. The statement "with H_2O" indicates the occurrence of the nucleophilic addition of a water molecule in the reaction chain leading to the product. MQ indicates that the product contains a quinone group.*

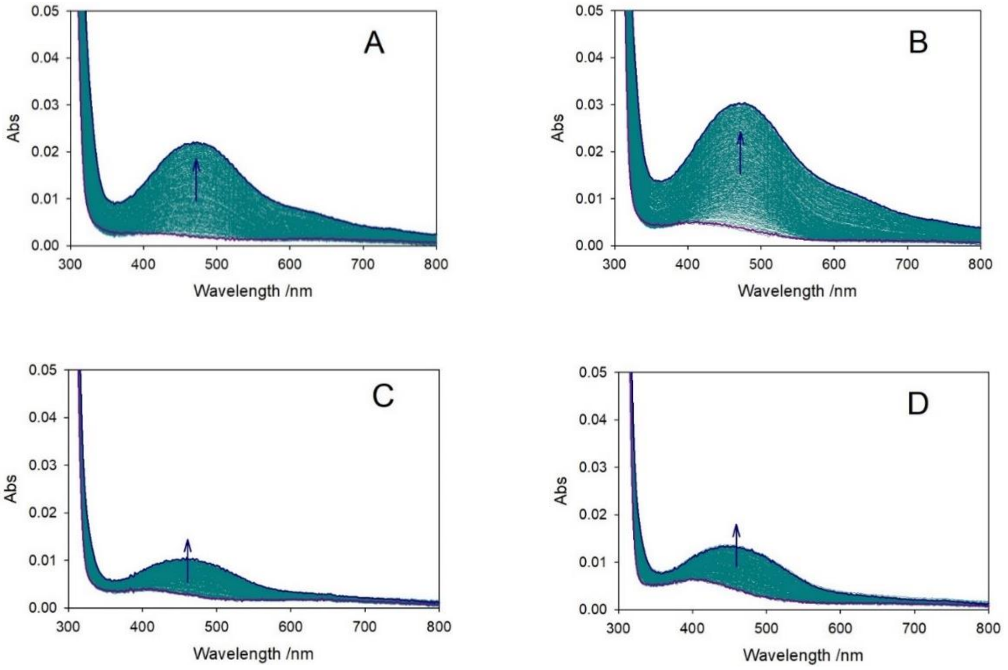

Figure S10. Absorbance changes with time during DA (0.3 mM) oxidation in 50 mM HEPES buffer solution at pH 7.4 and 20 °C in the presence of copper(II) (25 μM) and the following complexes (25 μM): Cu^{II}-NH_2-Aβ_{16} (panel A), Cu^{II}-Ac-Aβ_{16} (panel B), Cu^{II}-NH_2-Aβ_9 (panel C) and Cu^{II}-Ac-Aβ_9 (panel D).

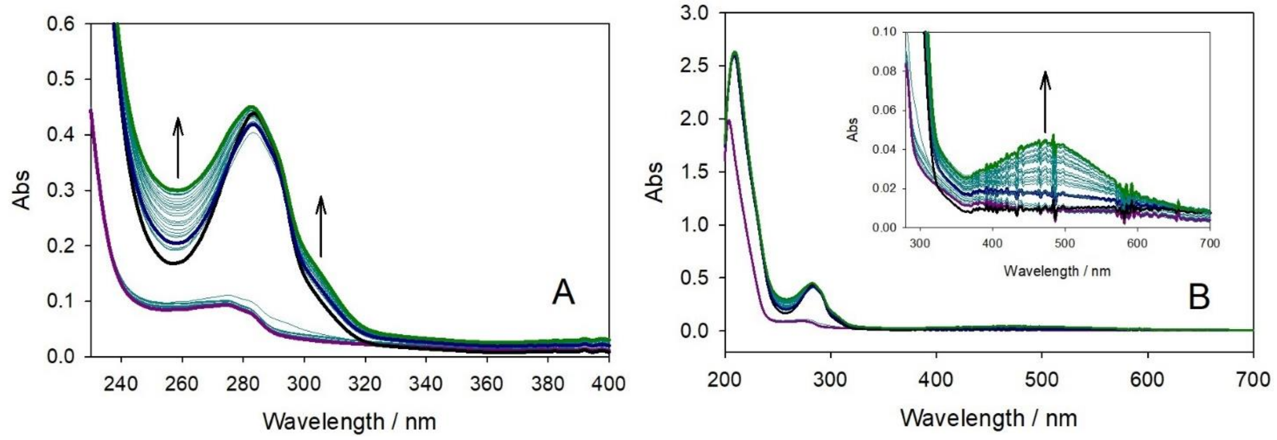

Figure S11. Absorbance changes with time of 4-chlorocatechol oxidation by Cu^{I}-Ac-Aβ_{16}. The complex was generated from an anaerobic solution of the peptide in 50 mM HEPES buffer at pH 7.4, upon vacuum/argon cycles, and addition of tetrakis(acetonitrile)copper(I) hexafluorophosphate (1:1, 25 μM) (violet spectrum). 4-Cholorocatechol (0.3 mM) was then added under argon atmosphere (black spectrum) and then the solution was exposed to 1 atm dioxygen and monitored for 15 min (blue spectrum shows the starting point while the green spectrum corresponds to the final acquisition). Panel A describes the absorbance variations in the region 230-400 nm, while panel B extends the spectrum to the visible region with an enlargement of the portion corresponding to the oxidation products of substrate.

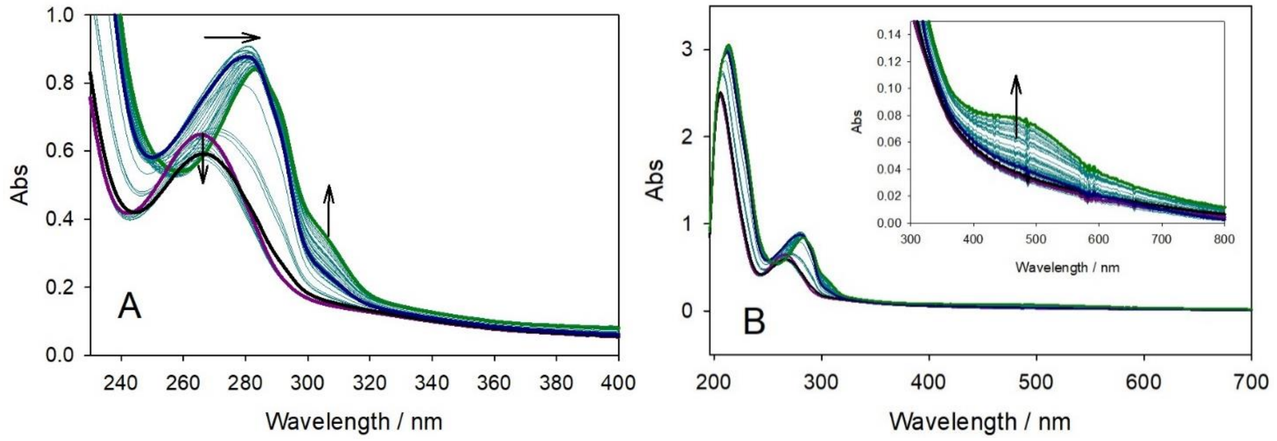

Figure S12. Absorbance changes with time of Cu^{I}-Ac-$A\beta_{16}$ complex (1:1, 25 μM) generated from an anaerobic solution of copper(II) and peptide in 50 mM HEPES buffer at pH 7.4 cycles and addition of ascorbate (2 equiv., 50 μM) (violet spectrum). 4-Cholorocatechol (0.3 mM) was added under argon atmosphere (black spectrum) and then the solution was exposed to 1 atm dioxygen and monitored for 15 min (blue spectrum shows the starting point while the green spectrum corresponds to the final acquisition). Panel A describes the absorbance variations in the region 230-400 nm, while panel B extends the spectrum to the visible region with an enlargement of the portion corresponding to the oxidation products of substrate.
